# Remimazolam for a patient with myotonic dystrophy type 1 who underwent endoscopic retrograde cholangiopancreatography under general anesthesia: a case report

**DOI:** 10.1186/s40981-021-00422-2

**Published:** 2021-02-24

**Authors:** Masakazu Fukuda, Shunsuke Tachibana, Noriaki Nishihara, Michiaki Yamakage

**Affiliations:** Department of Anesthesiology, Sapporo Medical University School of Medicine, South 1, West 16, Chuo-ku, Sapporo, Hokkaido 060-8543 Japan

**Keywords:** Remimazolam, General anesthesia, Myotonic dystrophy, Endoscopic retrograde cholangiopancreatography

## Abstract

**Background:**

Remimazolam is a benzodiazepine receptor agonist with an ultra-short-acting anesthetic effect. We used remimazolam for anesthesia in a patient with myotonic dystrophy type 1 who underwent endoscopic retrograde cholangiopancreatography (ERCP).

**Case presentation:**

A 58-year-old woman received ERCP under general anesthesia. She had impaired respiratory function due to myotonic dystrophy type I and was at a risk of respiratory complications after anesthesia. General anesthesia was induced with remimazolam 12 mg/kg/h, remifentanil 0.1 μg/kg/min and rocuronium 15 mg, followed by tracheal intubation and maintained with remimazolam 0.8−1.0 mg/kg/h. At the end of anesthesia, we injected sugammadex 150 mg and flumazenil 0.2 mg, allowing smooth and clear emergence from anesthesia. She was discharged from the hospital without any respiratory problems on postoperative day 5.

**Conclusions:**

Remimazolam was safe to use for general anesthesia in a patient with myotonic dystrophy type 1 undergoing ERCP.

## Background

Remimazolam was launched as a general anesthetic drug in August 2020 in Japan. As a benzodiazepine receptor agonist, remimazolam is an ultra-short-acting sedative/anesthetic and shows high affinity for γ-aminobutyric acid (GABA) receptors [1, 2]. The drug combines the safety profile of benzodiazepines regarding hemodynamic stability with fast onset and offset characteristics and improved controllability.

In this clinical case, we used remimazolam to safely manage a patient suffering from myotonic dystrophy type 1 during endoscopic retrograde cholangiopancreatography (ERCP) under general anesthesia.

## Case presentation

We obtained written informed consent from the patient for publication of this case report. The patient was a 58-year-old female (height 153 cm, weight 68 kg, BMI 29.0 kg/m^2^). Three years earlier, she had been definitively diagnosed with myotonic dystrophy type 1 by genetic test (CTG repeat 1100-2100). Symptoms of myotonic dystrophy included ax-like face, myotonia, peripheral muscle weakness and atrophy in the distal extremities, and proximal weakness in the lower extremities, and in terms of usual activities of daily livings, she was confined to a wheelchair. She also experienced repeated sputum congestion and aspiration, and her respiratory condition was classified as the equivalent of Hugh-Jones 5, caused by myotonic dystrophy. Preoperative pulmonary function tests revealed restrictive respiratory impairment (vital capacity (VC) 0.99L, %VC 35.9%, forced expiratory volume in the first second (FEV1.0) 103.2%), and an arterial blood gas analysis at room air showed pH 7.35, pCO_2_ 47.5 mmHg, and pO_2_ 65.5 mmHg. Furthermore, she used a continuous positive airway pressure device due to respiratory distress during night-time sleep. There were no abnormal findings on both the electrocardiogram and echocardiography.

This patient was newly diagnosed with gallstone pancreatitis with elevated amylase, common bile duct stones, and an enlarged main pancreatic duct on computed tomography and was admitted to our hospital. To prevent the patient’s exacerbation of respiratory failure and aspiration during treatment, ERCP under general anesthesia and perioperative management were scheduled.

Anesthesia was induced with remimazolam at 12 mg/kg/h and remifentanil at 0.1 μg/kg/min, and the endotracheal tube was inserted smoothly after administration of a total of 15 mg rocuronium. The rocuronium dose was determined by train-of-four (TOF) monitoring, and the patient was intubated in small doses of 5 mg each after the muscle twitches disappeared. Muscle relaxation monitoring was continued to minimize the administration of rocuronium during the surgery, total use of rocuronium was 20 mg. After induction of general anesthesia, we decreased the remimazolam dose to 1 mg/kg/h and maintained the remifentanil dose at 0.1 μg/kg/min. We attached the bispectral index (BIS) sensor to the patient's forehead and adjusted the maintenance dose of remimazolam assuming a target of BIS of 60–70 during anesthesia (0.8–1.0 mg/kg/h). Furthermore, to prevent a decrease in drug metabolism due to intraoperative hypothermia, we maintained the core body temperature over 36.0 °C by using forced-air warming system and blankets.

We chose the left lateral decubitus position during surgery. ERCP proceeded smoothly, administration of both remimazolam and remifentanil were stopped after ERCP ended. Spontaneous breathing resumed immediately, and we injected 150 mg of sugammadex after confirming a TOF count of 4/4. Awakening from remimazolam anesthesia was smooth, with eye-opening on the request of an anesthesiologist. However, at this stage, spontaneous breathing was weaker and tidal volume was smaller than before induction of anesthesia, 0.2 mg of flumazenil was used to antagonize and completely eliminate the muscle relaxant effect of remimazolam. The amount of sputum was such that we had to frequently perform intraoral and intratracheal suction, then extubation.

Operation time was 28 min, and total anesthesia time was 114 min (time until intubation, 5 min; time from end of surgery to extubation, 22 min). Modified Observer’s Assessment of Alertness/sedation score after extubation was 4. The patient was transferred to the ICU to monitor respiratory status, then was moved to the general ward the next day after no significant airway events were encountered postoperatively. On postoperative day 5, she was discharged from hospital without any respiratory complications.

## Discussion

Increased anesthetic risk has been observed in patients with myotonic dystrophy. In the perioperative period, evaluating the risk to myotonic dystrophy patients and selecting appropriate anesthetic drugs, opioids, and doses of neuromuscular blockade during surgery is important.

Mathieu et al. reported a perioperative complication rate of 8.2% and showed that the majority of them were found in the respiratory systems [3]. Using multivariate analysis, they also revealed that the risk of perioperative pulmonary complications was significantly higher after an upper abdominal surgery (odds ratio (OR) 24.4, 95% CI 4.0 to 149.3) and for patients with proximal limb weakness (OR 14.1, 95% CI 1.5 to 134.4).

The patients was predicted to be at high risk. In fact, the patient was constantly in a poor respiratory condition and symptoms were classified as grade 4 in the muscular impairment rating scale (grade 4, “mild to moderate proximal weakness”) [4], an elevated risk of postoperative respiratory complications was suggested from the preoperative stage. Moreover, if in non-surgical cases, we experienced a case with undiagnosed myotonic dystrophy who developed a sudden onset of dyspnea and required long-term respiratory management [5], and we need to be aware of patients with myotonic dystrophy type 1, including the possibility of sudden clinical changes.

In our clinical case, we used remimazolam followed by its antagonist flumazenil to prevent postoperative respiratory complications. Moreover, this case report is the first to present the use of remimazolam for general anesthesia in an ERCP patient with myotonic dystrophy type 1.

Remimazolam is a benzodiazepine drug, similar to midazolam, and has a mild muscle relaxant effect. This negative effect may exert adverse events such as respiratory depression and paralysis especially in patients with myotonic dystrophy. On the other hand, the advantages of remimazolam include less circulatory depression [6], fewer injection-site reactions during administration, and the availability of flumazenil to reverse the sedative effects. Remimazolam is also an ultra-short-acting intravenous anesthetic that is rapidly metabolized, primarily by hepatic tissue esterases, and its metabolites lack any activity, representing another benefit [2, 7]. These characteristics of remimazolam make the agent effective and safe for a wide range of patients undergoing general anesthesia, including the elderly, patients with unstable circulation and patients with respiratory failure. Doi et al. also reported that the safety and efficacy of remimazolam for inducing and maintaining general anesthesia, even in high-risk surgical patients (ASA-PS ≥ 3) [8]. Half of the participants showed comorbidities such as cardiovascular, metabolic, and respiratory failures. We speculated that no patients in that study were suffering from neuromuscular disease, and the use of remimazolam for such patients remains unverified.

Several studies on remimazolam as a sedative have been published and the efficacy of remimazolam has been introduced. Chen et al. demonstrated that remimazolam could provide safe, effective sedation for patients undergoing upper gastrointestinal endoscopy [9]. The present study suggests that remimazolam allows rapid recovery from sedation and has lower potential to cause cardiovascular and respiratory depression compared with propofol. In another study, Chen et al. also demonstrated that hypotension and respiratory depression as adverse events were decreased in a remimazolam group compared to a propofol group for colonoscopy [10]. As noted in these reports about remimazolam use, during the induction and maintenance of anesthesia, blood pressure was stable with no problems. Intraoperatively, we maintained BIS stability at around 60. After remimazolam administration was ended, the patient immediately emerged from anesthesia and the use of flumazenil improved the quality of awakening. Sinclair et al. demonstrated that a muscle relaxant without reversal was an independent risk factor in children with myotonic dystrophy [11]. This study was limited to pediatric cases; the conclusions might be different compared to adult ones. Remimazolam is not a muscle relaxant but does have a mild muscle relaxant effect; we judged to use flumazenil to antagonize remimazolam effect fully. Postoperative respiratory complications thus did not appear to arise in this case.

Verification of the effects of remimazolam and of those cases requiring flumazenil antagonism is necessary, along with an investigation of appropriate cases for remimazolam use in the future. However, at least in this clinical case, stable anesthesia management was possible, and postoperative respiratory complications were not encountered using remimazolam.

## Conclusion

Remimazolam may be safe to use in patients with myotonic dystrophy and other respiratory problems. We believe that we have identified another option for the anesthesia management in patients with respiratory problems.

## Data Availability

Not applicable due to patient privacy concerns.

## Abbreviations

GABA: Binding site γ-aminobutyric acid
ERCP: Endoscopic retrograde cholangiopancreatography
VC: Vital capacity
FEV1.0: Forced expiratory volume in the first second
TOF: Train of four
ICU: Intensive care unit
OR: Odds ratio
